# Epigenetic Reprogramming and Emerging Epigenetic Therapies in CML

**DOI:** 10.3389/fcell.2019.00136

**Published:** 2019-07-17

**Authors:** Jane Bugler, Ross Kinstrie, Mary T. Scott, David Vetrie

**Affiliations:** Wolfson Wohl Cancer Research Centre, Institute of Cancer Sciences, University of Glasgow, Glasgow, United Kingdom

**Keywords:** chronic myeloid leukemia, epigenetics, stem cells, therapies, drug resistance

## Abstract

Chronic myeloid leukemia (CML) is a hematopoietic stem cell disorder characterized by BCR-ABL1, an oncogenic fusion gene arising from the Philadelphia chromosome. The development of tyrosine kinase inhibitors (TKIs) to overcome the constitutive tyrosine kinase activity of the BCR-ABL protein has dramatically improved disease management and patient outcomes over the past 20 years. However, the majority of patients are not cured and developing novel therapeutic strategies that target epigenetic processes are a promising avenue to improve cure rates. A number of epigenetic mechanisms are altered or reprogrammed during the development and progression of CML, resulting in alterations in histone modifications, DNA methylation and dysregulation of the transcriptional machinery. In this review these epigenetic alterations are examined and the potential of epigenetic therapies are discussed as a means of eradicating residual disease and offering a potential cure for CML in combination with current therapies.

## Introduction

Chronic myeloid leukemia (CML) is a rare clonal hematopoietic stem cell disorder, with an annual incidence varying from 0.6 to 2 cases per 100,000 individuals (Rohrbacher and Hasford, 2009). CML is characterized by a genetic abnormality, termed the Philadelphia chromosome (Nowell and Hungerford, 1961), caused by a reciprocal translocation between the long arms of chromosomes 9 and 22, t(9;22)(q34;q11) (Rowley, 1973). This leads to the fusion of the BCR and ABL genes and the resultant BCR-ABL1 fusion protein, with its constitutive tyrosine kinase activity (Daley et al., 1990), transforms a hematopoietic stem cell (HSC) into a leukemic stem cell (LSC). The oncogenic BCR-ABL1 protein can activate multiple signaling pathways including RAS/RAF, PI3K/Akt, JUN kinase, and STAT which cause malignant transformation and drive the development of CML. This has been extensively reviewed previously (Deininger et al., 2000; Ren, 2005). Additionally, there are also multiple signaling pathways subverted in CML LSCs that promote their survival specifically (reviewed elsewhere; Holyoake and Vetrie, 2017).

Chronic myeloid leukemia is a tri-phasic disease consisting of a chronic phase (CP), accelerated phase (AP), and lymphoid or myeloid blast phase (BP) (Baccarani et al., 2013). At diagnosis, patients in CP have typically <10% blast cells (immature undifferentiated progenitors) in their peripheral blood and bone marrow, and their blood cells remain differentiated and minimally invasive (Faderl et al., 1999). Due to the complexity of the disease, progression varies between patients, with some progressing to more advanced stages within a few months and others remaining in CP for many years. Generally, if left untreated, the natural history of the disease is for the vast majority of cases to present in CP, with progression to AP and then to BC within 5 years (Giralt et al., 1995). In AP, there are increased numbers of blast cells in the blood and bone marrow (typically 15–29%), >20% basophils in the peripheral blood and bone marrow, persistent thrombocytopenia, and clonal chromosome abnormalities in Ph^+^ cells (Kantarjian et al., 1988, 1993; Baccarani et al., 2013). BP is characterized by the rapid expansion of a population of either myeloid or lymphoid blast cells to >30% of the peripheral blood and bone marrow, and extramedullary blast proliferation (Kantarjian et al., 1987; Baccarani et al., 2013). Patients in BP have a very poor prognosis and a reported median survival rate of 3–6 months (Kantarjian et al., 1987).

The treatment of CML was revolutionized by the introduction of the TKI, imatinib, in 1996 which displays significant anti-leukemic effects and can target CML cells in peripheral blood and bone marrow (Druker et al., 1996, 2001). Since the introduction of TKIs into the clinic, the number of patients achieving a major molecular response has dramatically increased (O’Brien et al., 2003), and the development of imatinib was followed by second and third generation TKIs such as dasatinib (Lombardo et al., 2004; Shah et al., 2004), nilotinib (Weisberg et al., 2005; Kantarjian et al., 2006), bosutinib (Khoury et al., 2012), and ponatinib (O’Hare et al., 2009). Unsurprisingly, the prevalence of CML has increased due to the introduction of TKIs as the main form of treatment, making CML a manageable, chronic disease (Rohrbacher and Hasford, 2009).

However, whilst TKI therapy has transformed the treatment of CML, 25–30% of CP-CML patients fail TKI therapy, where half of these cases have mutations in the BCR-ABL1 kinase domain (Baccarani et al., 2013), while the reason for failure in the remaining 50% of patients is unclear. Residual BCR-ABL1^+^ progenitor cells have been consistently detected in patients who have responded well to TKI and achieved complete cytogenetic responses (Bhatia et al., 2003), demonstrating that the disease persists in patients despite long-term TKI treatment (Chomel et al., 2011, 2016; Chu et al., 2011). It is a widely held view that this minimal residual disease is maintained by the survival of a sub-population of LSCs in the bone marrow (Holyoake et al., 1999; Chomel et al., 2011; Chu et al., 2011). Approximately 60% of CP-CML patients who respond well to TK have an LSC persistence phenotype. Whilst 10–20% of patients who achieve deep molecular responses following TKI therapy can discontinue treatment, half of these patients will have disease recurrence with 12 months – further supporting the presence of persisting LSC in residual disease (Mahon et al., 2010; Ross et al., 2013).

Due to the low cure rate and risk of disease recurrence and progression, understanding the mechanisms that underpin CML cell survival is integral to identifying novel drug targets and developing new treatments for the disease. A number of pathways that the LSCs use for survival have been studied and reviewed elsewhere (Holyoake and Vetrie, 2017), and novel therapies that target some of these are currently being developed. Here, we will focus on the evidence for epigenetic dysregulation and re-programming in CML, and its relevance to developing novel therapeutic strategies.

## The Polycomb Complexes in HSC and CML LSC

Since the first links between cancer and epigenetic reprogramming were established in 1983 (Feinberg and Vogelstein, 1983), mounting evidence suggests that cancers are driven by both genetic and epigenetic alterations, and some of these alterations may precede the development of frank leukemia as a pre-leukemic states (see below). Furthermore, the consequences of epigenetic reprogramming may have a greater influence in stem cells, as many epigenetic processes are required for stem cell maintenance and embryonic development (Feinberg et al., 2006; Avgustinova and Benitah, 2016a,b). Pertinent to CML, the acquisition of the BCR-ABL1 mutation not only transforms the HSC to an LSC, but it also drives epigenetic reprogramming. One group of epigenetic regulators that are known to be deregulated in CML LSC are the Polycomb-group (PcG) proteins. These proteins are involved in gene silencing and have been shown to play an essential role in development, stem cell biology and differentiation, and are particularly important in hematopoiesis (Di Carlo et al., 2019). The PcG proteins comprise two complexes: Polycomb Repressive Complex 1 and 2 (PRC1 and PRC2).

### PRC2 Complex

PRC2 is responsible for methylation of histone H3 on lysine 27 (H3K27), one of the main features of silenced chromatin. In mammals, PRC2 is composed of three core components, an Enhancer of Zeste ortholog (EZH1 or EZH2), Suz12 and an isoform of EED (EED1–4). The EZH proteins, which have a conserved SET domain, are responsible for the histone methyltransferase activity of the complex, while SUZ12 and EED allow binding to the nucleosome. In addition, the complex can contain a number of other cofactors that include JARID2, PHF1, PHF19, RBBP5, and RBBP7 with recent evidence suggesting that JARID2, which contains a DNA binding site, may play a role in the activity and recruitment of PRC2 (Shen et al., 2009; Herz and Shilatifard, 2010; Li et al., 2010). EZH1-PRC2 and EZH2-PRC2 complexes are mutually exclusive but as a general rule EZH2-PRC2 appears to be responsible for global H3K27 di- and tri-methylation, while EZH1-PRC2 has weaker activity and mediates H3K27 mono- and di-methylation (Margueron et al., 2008; Hidalgo et al., 2012).

Studies into the role of PRC2 components in HSC have been somewhat contradictory. While gain-of-function studies suggest that EZH2 is an important regulator of self-renewal (Kamminga et al., 2006; Herrera-Merchan et al., 2012), knockout of EZH2 in mice had no effect on the HSC compartment, although B and T cell development were affected (O’Carroll et al., 2001). Indeed further reports suggest EZH2, EED and SUZ12 have inhibitory effects on HSCs (Lessard et al., 1999; Majewski et al., 2008). While the reason for these conflicting results is unclear, a recent report has shown that in adult HSCs, EZH1 can compensate for EZH2 loss (Mousavi et al., 2012) which may help to explain these discrepancies. Indeed, while loss of EZH2 had no effect on HSCs, EZH1 was found to be essential for adult HSC maintenance, with loss leading to senescence (Hidalgo et al., 2012). Here EZH1 was shown to be responsible for mono- and di-methylation of H3K27 which may be required for EZH2 and PRC1 activity. Clearly the composition of the PRC2 complex and the levels of each of its components plays a key role in its activity.

**FIGURE 1:**
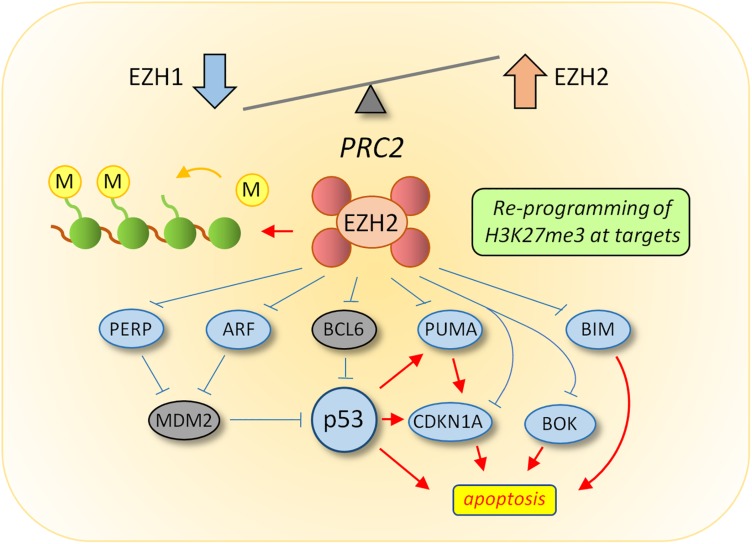
Reprogramming of PRC2 in CML LSC. Transcriptomic analysis revealed an altered balance between EZH1 and EZH2 in LSC, the consequence of which results in re-programming of H3K27me3 targets to suppress apoptosis. Upstream activators of p53 activity (ARF and PERP) and pro-apoptotic downstream targets of p53 (BIM, BOK, and PUMA) were all targets of H3K27me3-mediated repression in LSC. Current data supports a model whereby EZH2 inhibitors induce apoptosis in CML LSC through the up-regulation of EZH2 targets upstream of p53 (such as ARF), which could lead to increased p53 levels, or through up-regulation of p53 target genes directly which are normally repressed by EZH2 activity.

EZH2 has been shown to be upregulated in multiple solid tumors, including breast, ovarian, pancreas, prostate and lung, and is often associated with poor prognosis (Crea et al., 2012). In hematological malignancies, the situation is somewhat more complicated as EZH2 appears to have both oncogenic and tumor suppressor activities (Lund et al., 2014). While no mutations in the PRC2 proteins have been identified in CML, a number of studies using cell lines, primary cells and mouse models have shown that the expression of PRC2 components are dysregulated (Nishioka et al., 2016; Scott et al., 2016; Xie et al., 2016). In CML LSCs from chronic phase patients, EZH1 expression levels were low relative to normal HSCs, while EZH2 and a number of co-factors including Suz12, Jarid2, and PHF19 were all upregulated (Scott et al., 2016). This was shown to be associated with a reprogramming of H3K27me3 at PRC2 target genes, resulting in altered dependency on EZH2 for survival in CML LSCs compared to normal cells (Scott et al., 2016; Xie et al., 2016; Figure 1). Interestingly, recent evidence from Agarwal et al. (2019) demonstrates that CXCL12 levels in mesenchymal stromal cells (MSCs) may influence EZH2 and EZH1 expression levels in CML stem cells indicating a role for the bone marrow microenvironment (BMM) on this effect. In addition, delay of induction of CML in an EZH2 knock out mouse model suggests EZH2 is also required for initiation of the disease (Xie et al., 2016). Whether this means that pre-leukemic epigenetic reprogramming of PRC2 is required for disease initiation has yet to be established.

Given the increasing evidence for a role of PRC2 in cancer, unsurprisingly a number of therapeutics targeting this complex have emerged (Fioravanti et al., 2018) and are now in Phase I and II clinical trials, including diffuse large cell B-lymphoma (DLBCL), follicular lymphoma (FL), solid tumors, and multiple myeloma (Gulati et al., 2018). Using a patient derived xenograft model of CML, Scott et al. (2016) were able to demonstrate significant targeting of CML stem cells with the EZH2 inhibitor Tazemetostat in combination with TKI compared to TKI treatment alone. This suggests that combined treatment may represent a novel therapeutic approach for treatment of CML. Indeed a new Phase II clinical trial in relapsed and refractory CML – TASTER (soon to be recruiting patients) – will include Tazemetostat as one of the arms.

### PRC1 Complex

PRC1 is responsible for laying down ubiquitination on histone H2A lysine residue K119. In the canonical model of PRC1 and PRC2 function, PRC1 binds to chromatin through H3K27me3 and ubiquitinates H2AK119 through the action of the RING1A or RING1B E3 ubiquitin ligases which are the catalytic components of the complex (Buchwald et al., 2006; Li et al., 2006). This allows compaction of the chromatin causing further repression of gene transcription. In addition to the RING finger E3 ligases, the canonical PRC1 complex contains a number of other core components, including Polycomb group zinc finger (PCGF) proteins which bind to and stabilize RING1A/1B. BMI1 (PCGF4) is also a member of this group and has been shown to have a key role in both normal and leukemic HSCs (Rizo et al., 2008, 2009; Schuringa and Vellenga, 2010) along with other stem cells and CSCs (Sangiorgi and Capecchi, 2008; Chatoo et al., 2010; Facchino et al., 2010; Allegra et al., 2014; Zhu et al., 2014; Srinivasan et al., 2017; Yanai et al., 2017). While knock out of BMI1 suppressed self-renewal in normal HSC, overexpression enhanced this process (van der Lugt et al., 1994; Park et al., 2003; Iwama et al., 2004) and resulted in long-term maintenance of human haemopoietic stem and progenitor cells (Rizo et al., 2008). BMI1 is upregulated in multiple tumors including lymphomas (Bea et al., 2001) prostate (Goel et al., 2012), breast (Kim et al., 2004a), colon (Kim et al., 2004b), and non-small cell lung cancer (NSCLC) (Vonlanthen et al., 2001), as well as in hematological malignancies such as acute myeloid leukemia (AML) and myelodysplastic syndrome (MDS) (Saudy et al., 2014).

In normal hematopoiesis, BMI1 expression is highest in HSC and decreases as cells mature. In CML however, compared to normal HSCs, chronic phase CD34^+^ cells have increased expression of BMI1 and the levels continue to increase with disease severity through accelerated phase and blast phase, with high expression levels correlating with poor prognosis (Mohty et al., 2007; Saudy et al., 2014). This suggests a role for BMI1 in CML development. Indeed, co-expression of BMI1 and BCR-ABL in normal CD34^+^ cells led to transplantable leukemia in immunosuppressed mice (Rizo et al., 2010). However, while overexpression of BMI1 in a CML lymphoid progenitor cell resulted in development of B-ALL in a mouse model of CML, expression of BMI1 in a CML HSC did not result in a serially transplantable disease (Sengupta et al., 2012).

The increase in BMI1 expression in chronic phase CML CD34^+^ cells correlated with a decrease in the expression of CCNG2 (cyclin G2), leading to an inhibition of autophagy. Targeting BMI1 in these cells by either knock down or inhibition with one of the BMI1 inhibitors that have now been developed led to a decrease in clonogenic survival, suggesting therapeutic potential of BMI1 inhibition. Combination with TKI in CP-CML however elicited no further reduction in survival (Mourgues et al., 2015). Given the obvious functional links between PRC1 and PRC2 and the effect that has already been shown on LSC survival by EZH2 inhibition (Scott et al., 2016), perhaps targeting both BMI1 and EZH2 in parallel could have therapeutic potential in CML. Indeed, dual inhibition of EZH2 and BMI1 has already been shown to have more pronounced effects *in vitro* and *in vivo* in glioma stem cells than either agent alone (Jin et al., 2017) and in multiple myeloma (Alzrigat et al., 2017).

## HDAC and HAT Reprogramming

Alterations of histone and non-histone acetylation occur widely in cancer (Archer and Hodin, 1999) due to the opposing effects of histone acetyltransferases (HATs) and histone deacetylases (HDACs). It is well established that changes in the expression and activity of HATs and HDACs disrupt the balance between acetylation and deacetylation, and can promote leukemogenesis (Gao et al., 2013; Ahmadzadeh et al., 2015).

### SIRT1

Sirtuin 1 (SIRT1) is a NAD-dependent HDAC upregulated in CD34^+^ CML cells compared to normal hematopoietic progenitors and has been implicated in leukemogenesis and the survival of CML LSCs through its activity on a non-histone target p53 (Li et al., 2012; Yuan et al., 2012). SIRT1 is activated by STAT5-mediated binding to the SIRT1 promoter, and consequently loss of STAT5 *in vitro* reduced SIRT1 promoter activity (Yuan et al., 2012). SIRT1 activation resulted in the deacetylation of a number of targets including p53, negatively regulating p53 transcriptional activity, and promoting CML cell survival (Li et al., 2012; Yuan et al., 2012; Chen and Bhatia, 2013). Furthermore, SIRT1 has been implicated in promoting genetic instability of CML cells through deacetylation of components of the DNA repair machinery and thus increasing the incidence of error-prone DNA repair (Wang et al., 2013). SIRT1 expression promoted the acquisition of BCR-ABL mutations and SIRT1 knockdown supressed genetic mutations of hypoxanthine phosphoribosyl transferase (HPRT) in the KCL22 CML cell line, suggesting that inhibition of SIRT1 may be able to overcome drug resistance. SIRT1 has also been implicated in promoting autophagy in CML cells, through the deacetylation of LC3, allowing it to associate with other autophagy factors and localize to the cytoplasm (Huang et al., 2015).

Following imatinib treatment in CML cell lines, SIRT1 expression was decreased, but not completely depleted (Yuan et al., 2012), providing a rationale for direct SIRT1 inhibition. Both genetic knockdown and pharmacological inhibition of SIRT1 in CML CD34^+^ cells resulted in the acetylation and activation of p53, and subsequent upregulation of downstream pro-apoptotic factors (Li et al., 2012). This resulted in the decreased proliferation and enhanced apoptosis of CD34^+^ CML cells, as well as the selective killing of CML LSCs *in vitro* and *in vivo*. Treatment with the SIRT1 inhibitor also enhanced the effect of TKI treatment via activation of p53 signaling (Li et al., 2012; Chen and Bhatia, 2013). Moreover, SIRT1 has been implicated in the process of aging, where epigenetic silencing of HIC1 upregulates SIRT1 expression (Chen et al., 2005). Therefore, the inhibition of SIRT1 may have more clinical relevance in older patients, although, to date, this hasn’t been explored. SIRT1 inhibitors have been tested in Phase I and II clinical trials for a number of disorders, exhibiting good safety and efficacy profiles (Hoffmann et al., 2013; Sussmuth et al., 2015; van der Meer et al., 2015).

### Other HDAC and HAT Activities

Following the acquisition of BCR-ABL in CML cells, HDAC1 is relocalised to the cytoplasm, where its function is depleted (Brusa et al., 2006). Consequently, this results in hyperacetylation of histone H4 at the BCR-ABL promoter region – further reinforcing its transcription. Interestingly, treatment with imatinib resulted in the restoration of nuclear HDAC1 in primary CD34^+^ cells and a reduction in BCR-ABL1 transcript levels, which correlate with histone H4 deacetylation (Brusa et al., 2006). Another study demonstrated increased lysine 317 (K317) acetylation of p53 mediated by BCR-ABL1, which regulates post-translational p53 activity (Kusio-Kobialka et al., 2012). BCR-ABL1-dependent acetylation prevented the translocation of p53 to the cytoplasm where it engages in p53/Bax-mediated mitochondrial-dependent apoptosis in response to DNA damage – thus further establishing a link between increased acetylation and CML cell survival. However, paradoxically, treatment with various HDAC inhibitors, which induce acetylation, also result in decreased survival of CML cells (Nimmanapalli et al., 2003a,b; Fiskus et al., 2006a,b). Further examination of the effects of HDAC inhibitors with imatinib on primitive LSCs resulted in increased apoptosis of the CML progenitors (Zhang et al., 2010). Moreover, the HDAC inhibitor SB939 could overcome the deletion of the pro-apoptotic factor BIM (BCL-2 like 11), which is associated with imatinib-resistance (Ng et al., 2012), inducing apoptosis in CML cells harboring a BIM deletion (Rauzan et al., 2017). Overall, the data suggests that the role of histone and non-histone acetylation is complex in CML, and that therapeutic interventions that increase or decrease acetylation in CML cells may have clinical benefit.

## Epigenetic Reprogramming Following TKI Therapy

Whilst TKI therapy has revolutionized the treatment of CML and resulted in increased progression-free survival for patients in CP-CML, TKI does not eradicate all CML cells and a population of TKI-persistent LSCs survive and are responsible for disease recurrence (Holyoake et al., 1999; Bhatia et al., 2003; Copland et al., 2006). Of the cells that survive TKI therapy, a number of genetic and epigenetic changes have been reported which alter their survival mechanism and downstream signaling pathways (reviewed further in Holyoake and Vetrie, 2017). Pathways that are modified following TKI therapy may be examined as potential therapeutic targets to eradicate the survival of TKI-persistent LSCs.

### BCL6

BCL6 is a transcription factor commonly mutated in lymphoma cells where it can epigenetically regulate a number of its targets through modifications in chromatin accessibility at promoter and enhancer regions (Hatzi et al., 2013). Whilst its role in CML is not well characterized, BCL6 is expressed at low levels in TKI-naïve CML cells, however, its levels are significantly upregulated following TKI treatment in CML cell lines and primary CD34^+^ cells (Hurtz et al., 2011; Madapura et al., 2017). This is thought to occur through the re-activation of its upstream activator, FOXO3a, a member of the FOXO (forkhead box) family of transcription factors, following treatment with TKI (Brunet et al., 1999; Komatsu et al., 2003; Pellicano et al., 2014). As BCL6 directly represses the expression of its targets involved in cell cycle and DNA damage, including p53, upregulation of BCL6 has been shown to contribute to its anti-apoptotic phenotype (Phan and Dalla-Favera, 2004; Hurtz et al., 2011; Pellicano and Holyoake, 2011). Similar observations were made in Ph^+^ ALL, where BCL6 expression was upregulated following TKI treatment, resulting in the repression of CDKN1A, CDKN1B, and TP53 (Duy et al., 2011).

Mechanistically, BCL6 can epigenetically regulate its targets through complexes where it interacts with its corepressors BCOR, NCOR1, and SMRT, or directly via its RD2 or zinc finger domains. These complexes require the recruitment of class I and II HDACs to BCL6 recognition sites, resulting in chromatin remodeling and gene regulation (Wong and Privalsky, 1998; Lemercier et al., 2002). In hematopoiesis, BCL6 interacts with the Mi-2/NuRD (nucleosome remodeling deacetylase) complex, an abundant deacetylase complex involved in chromatin remodeling (Denslow and Wade, 2007). The Mi-2/NuRD complex is responsible for maintenance of HSCs and lineage differentiation during hematopoiesis, and conditional deletion of Mi-2β in the bone marrow resulted in the loss of both lymphoid and myeloid lineage cells (Yoshida et al., 2008). Furthermore, BCL6 regulates NOTCH signaling in neural cells, repressing NOTCH downstream targets through the recruitment of SIRT1 at promoter regions of target genes (Sakano et al., 2010; Tiberi et al., 2012). However, the relationships between BCL6 and its potential interacting partners have yet to be explored in CML, but this data provides a rationale for examining the links between BCL6 and histone deacetylases – both of which are survival factors in CML.

**FIGURE 2:**
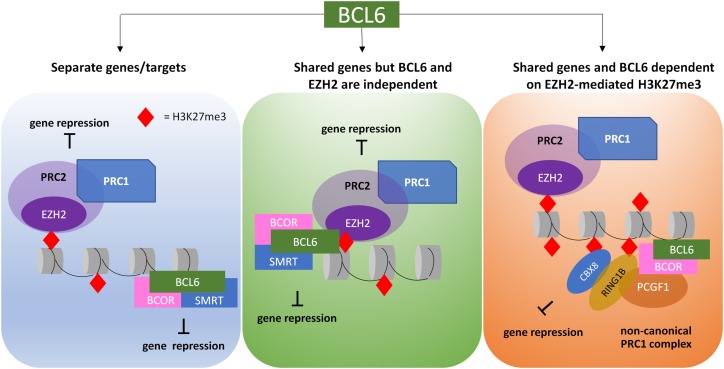
Proposed models of crosstalk between EZH2/H3K27me3 targets and BCL6 targets in CML LSC. PRC2, through interaction with PRC1, is able to promote gene repression via the deposition of H2AK119 ubiquitylation (H2AK119ub) and H3K27 trimethylation (H3K27me3). BCL6 recruitment can result in either H2AK119ub or protein and/or histone deacetylation, resulting in gene repression. Activities of PRC2 and BCL6 can be directed at different genes or the same genes; in the latter, the protein complexes may work independently or dependently, thus resulting in different outcomes when treated with EZH2 or BCL6 inhibitors.

Of particular interest in CML is the interaction of BCL6 with the PcG proteins. In lymphoma, BCL6 is involved in epigenetic reprogramming of its targets through interacting with both PRC1 and PRC2 complexes. H3K27me3 forms a binding site for CBX8, a component of non-canonical PRC1, allowing the BCL6 corepressor BCOR to be recruited. BCL6 then forms a ternary complex with BCOR and SMRT mediating repression at gene promoters marked with H3K27me3 by the PRC2 enzyme EZH2 on bivalent chromatin (Hatzi et al., 2013; Beguelin et al., 2016). This relationship is further demonstrated through the recruitment of PcG proteins and H2A ubiquitination by BCOR to BCL6 targets (Gearhart et al., 2006). In CML CD34^+^ cells, EZH2 inhibition plus TKI upregulated a number of BCL6 targets such as p53, despite BCL6 upregulation, suggesting that BCL6 and EZH2 share a number of targets, and that BCL6 mediated repression may be dependent on EZH2-PRC2 (Hurtz et al., 2011; Beguelin et al., 2016; Scott et al., 2016). The proposed mechanisms through which BCL6 and EZH2 may interact in CML cells is shown in Figure 2, and provides a rationale for combined BCL6 and EZH2 inhibition in CML cells.

BCL6 inhibition has been investigated in CML cells as a novel approach to eradicate LSCs (Hurtz et al., 2011). A number of inhibitors have been developed over the past decade that are specific to BCL6 activity and target the BCL6 lateral groove resulting in the disruption of corepressor binding and the re-activation of BCL6 target genes. Combined treatment of the BCL6 peptide inhibitor reteroinverso-BCL6 peptide inhibitor (RI-BPI) with imatinib prevented TKI resistance and potentiated the effects of imatinib observed in Ph^+^ ALL (Cerchietti et al., 2009; Duy et al., 2011). Furthermore RI-BPI enhanced the effects of imatinib treatment in CD34^+^38− CML LSCs, as well as decreasing primary CML cell colony forming capacity, and eradicated CD34^+^38− LSCs through increased apoptosis (Hurtz et al., 2011). Other BCL6 small molecule inhibitors, 79-6 (Cerchietti et al., 2010) and FX1 (Cardenas et al., 2016) have also been developed, and FX1 in combination with TKI significantly decreased colony-forming capacity of CML CD34^+^ cells (Madapura et al., 2017).

### DNA Methylation

CD27 and its ligand CD70 has been implicated in CML LSC survival through their regulation of Wnt signaling (Schurch et al., 2012), a critical pathway in stem cell self-renewal (Zhao et al., 2007). The mechanism by which this occurs is complex, dependent on TKI treatment, and involves down-regulation of microRNA-29 and changes in DNA methylation (Riether et al., 2015). DNMT1A is positively regulated by microRNA-29 isoforms a/b, and TKI treatment results in down-regulation of DNMT1A. However, SP1 is up-regulated upon TKI treatment as microRNA-29 isoform c is a negative regulator of SP1. Hypomethylation of the CD70 promoter facilitates binding of SP1 which up-regulates CD70, stimulates CD27-mediated signaling through the Wnt pathway, which promotes resistance of LSC to TKI (Nolte et al., 2009). Antibody-based inhibition of the CD70/CD27 interaction in combination with TKIs significantly targeted the CD34^+^ CML stem/progenitor cells *in vitro* and *in vivo*. Furthermore, CD70/CD27 may be associated with a more aggressive CML phenotype, as CD70/CD27 was upregulated in AML blast cells and progenitors, and increased levels of soluble CD27 is used as a prognostic biomarker for poor overall AML survival (Riether et al., 2017).

BCL2-like protein (BIM) is an apoptotic activator, and has been shown to be epigenetically reprogrammed following treatment with TKIs. While early studies following TKI treatment showed that imatinib activated the BH3-only proteins BIM and BMF transcription post-translationally (Essafi et al., 2005; Kuroda et al., 2006), more recent evidence demonstrates that following TKI treatment BIM levels are downregulated and associated with decreased optimal responses (San Jose-Eneriz et al., 2009a). This occurs via DNA methylation of the BIM promoter which was observed in two different CML cell lines (BV173 and KU812). Hypermethylation in 36% of patients in CP-CML correlated with decreased BIM expression, and was associated with poor response to imatinib. A combination of imatinib with 5-aza-deoxycytidine, a demethylating agent, induced the expression of BIM and decreased cell proliferation and viability of CML cell lines (San Jose-Eneriz et al., 2009a). Intriguingly, chromatin immunoprecipitation (ChIP) experiments revealed the BIM gene promoter region was hypomethylated despite DNMT1 and EZH2 binding to this site (Bozkurt et al., 2013a). However, more recent evidence using ChIP-seq has shown that the BIM gene is associated with H3K27me3 in primary patient samples (Scott et al., 2016; Figure 1), suggesting that repression of BIM can be facilitated in multiple ways in CML.

## Non-Coding RNAs

Non-coding RNAs (ncRNAs), as their name suggests, are not translated into proteins, but they do regulate mRNA levels and protein translation through transcriptional interference. MicroRNAs (miRNAs), in particular are short strands of ncRNAs (∼20–23 nucleotides long) that can bind to specific sequences, most often the 3′ untranslated region (UTR) of target mRNA, preventing translation or cleaving the mRNA (He and Hannon, 2004). miRNAs can be regulated by BCR-ABL1, and have been demonstrated to play a role in the pathogenesis of CML (Machova Polakova et al., 2013). Moreover, miRNAs can directly target and regulate BCR-ABL1 expression. Furthermore, there are miRNA expression signatures which are used to distinguish between CML and normal cells (Agirre et al., 2008; Koschmieder and Vetrie, 2018), between clinical phases of CML (Machova Polakova et al., 2011), and between TKI responders and non-responders (San Jose-Eneriz et al., 2009b) (further reviewed in Machova Polakova et al., 2013; Kotagama et al., 2015; Di Stefano et al., 2016). Below, we describe a few examples of miRNAs that are mis-regulated in CML.

The expression of miR-150 and miR-146a are significantly decreased in CML cells at diagnosis (Agirre et al., 2008), and in advanced phases of CML (AP-CML and BP-CML) (Machova Polakova et al., 2011). Both miRNAs are regulated by BCR-ABL1, and their expression levels are restored following 2-week imatinib treatment (Flamant et al., 2010). Furthermore, miR-150 may be a useful biomarker for disease progression, where its lower expression correlates to poor prognosis and more advanced phases of CML (Kotagama et al., 2015). miRNA-203 negatively regulates the BCR-ABL1 mRNA, but it is epigenetically silenced in CML through the methylation of its promoter region (Bueno et al., 2008). The introduction of miR-203 to miR-203-deficient CML cell lines resulted in a decrease in BCR-ABL1 expression and subsequent decrease in CML cell proliferation (Bueno et al., 2008). One study demonstrated that TKI therapy upregulated 48 miRNAs, including miR-203, through inducing demethylation of miR-203 at its promoter region (Shibuta et al., 2013). These studies suggest that TKI may be able to restore the levels of some miRNAs and that this process may have a role in mediating the effect that TKIs have on CML cells.

## Epigenetic Mechanisms in CML Disease Progression

As described above, CML has three distinct phases. Due to the advent of TKIs, only 1-1.5% of CML cases will progress to blast phase usually due to resistance to TKI. Clinically, the aim is to induce a second chronic phase in patients who have progressed to advanced phase CML with increased dosing of TKI, to allow bone marrow transplantation. As TKIs are largely ineffective at this stage of disease, patients that progress to blast phase are typically treated similarly to those with AML and have a median survival of approximately 6 months (Perrotti et al., 2010). Therefore, more targeted therapies are warranted for these patients. Blast phase is typically characterized by the accumulation of additional chromosomal abnormalities (ACAs), but epigenetic regulation is also disrupted (reviewed also in Bozkurt et al., 2013b). While epigenetic changes occurring in CP-CML and in the progression to BP-CML have been studied extensively, changes occurring within the LSC compartment during disease progression are poorly understood and warrant further study. Research around this area is hampered due to the difficulty in isolating true BP-CML LSCs by immunophenotyping. Recent studies in patient-derived xenograft mouse models have shown that engraftable cells exist in all so-called stem and progenitor populations from BP-CML patient samples, usually a preserve of true HSCs in normal samples and CP-CML patient samples (Kinstrie et al., 2016).

### Mutations in Epigenetic Regulators

Using a combination of whole exome sequencing, copy number variation and RNA sequencing, a recent study identified clinically relevant variants of epigenetic regulators, ASXL1, SETD1B, IDH1, EZH2, and KMT2D at chronic phase diagnosis in patients with poor outcomes i.e., progression to blast phase (56% of 27 patients). In addition to these, mutations in PHF6, SETD2, and MLL (KMT2A) fusions were observed in all patients already in blast phase (Branford et al., 2018). Mutations in any of these genes at diagnosis of chronic phase were all associated with poorer outcome. ASXL1 has long been viewed as a potential predictor of CML evolution and mutations in this gene can be found in CP and BP CML and in both the CP and BP clones of patients who have progressed (Boultwood et al., 2010; Menezes et al., 2013). However, ASXL1 mutations conferred significantly slower progression to BP than other mutations and have even been associated with patients who went on to achieve MMR and did not have detectable ASXL1 variants at remission suggesting eradication of this clone (Branford et al., 2018). Interestingly, ASXL1 mutants through their interaction with BRD4 were hypersensitive to bromodomain inhibitors (Yang et al., 2018), providing a potential treatment avenue for patients with these mutations in CML. The overall findings of these sequencing-based approaches suggests that identifying mutations at diagnosis that are associated with poor outcome (i.e., failure to achieve MMR) or progression to blast phase may be helpful for assessing potential treatment options in addition to TKI therapy.

These sequencing-based studies take on added significance when one compares the mutations found in BP-CML to those identified in AML. It is now widely regarded that mutations in pre-leukemic clones (Jan et al., 2012) are the initial events leading to progression to frank AML. Studies have identified that the earliest mutations in AML occur most commonly in epigenetic regulators, in particular DNMT3A, TET2, and IDH1/2 followed by secondary mutations in genes associated with signal transduction pathways and cellular proliferation (Corces-Zimmerman et al., 2014; Shlush et al., 2014; Chotirat et al., 2015; Eriksson et al., 2015; Sato et al., 2016). This suggests that BP-CML may be facilitated by pre-leukemic lesions than precede the acquisition of BCR-ABL – particularly in cases where patients present with BP at diagnosis. Furthermore, whilst the study of pre-leukemic clones in AML is well advanced, this area is research is highly under-developed in CML, and warrants further attention, given that clinical grade compounds that target IDH1/2 (Popovici-Muller et al., 2018) are now available and have undergone clinical trials in AML.

### DNA Methylation and Progression

DNA methylation of CpG island regions is associated with gene silencing, and in malignancies these regions are frequently methylated resulting in the repression of genes that are associated with disease (Esteller, 2008). One of the most commonly cited explanations of CML evolution, progression and poor outcome is aberrant DNA methylation (Dunwell et al., 2010; Jelinek et al., 2011; Amabile et al., 2015) which is believed to promote defects in differentiation in AML cells (Lu et al., 2012; Corces et al., 2016). Methylation of genes associated with CML progression has been studied extensively over the years, identifying a number of candidates potentially involved in the process. Genes such as DAPK1 (Qian et al., 2009; Uehara et al., 2012; Celik et al., 2015), CALCA (Malinen et al., 1991; Nelkin et al., 1991; Mills et al., 1996), the cyclin dependent kinase inhibitors CDK2NA (Kusy et al., 2003; Nagy et al., 2003; Uehara et al., 2012), and CDK2NB (Kusy et al., 2003; Jelinek et al., 2011; Uehara et al., 2012) have been identified as hypermethylated by a number of investigators, but relatively little is still known about the overall impact of DNA methylation across the methylome in CML, and additionally in the progression to blast phase. More recently groups have tried to identify differences in methylation patterns in sorted CML cells vs normal to allow for more targeted treatments in chronic phase (Maupetit-Mehouas et al., 2018) and also by using reduced representation bisulfite sequencing (RRBS) and RNA sequencing to identify changes in methylation and gene expression as the disease progressed (Heller et al., 2016). When compared to control samples approximately 600 differentially methylated CpG sites were identified in patients with CP-CML. However, when BP-CML patients were analyzed, around 6500 were found to be differentially methylated and that in patients that progressed from CP to BP, 897 genes were methylated at time of progression, but not at diagnosis (Heller et al., 2016). Single nucleotide variants (SNV) in epigenetic modifiers have been identified in CML on a number of occasions. In CP-CML, DNMT3A, EZH2, RUNX1, and TET2 were found to be mutated (Schmidt et al., 2014), whilst in a separate study, BP-specific SNVs were found in TET2, ASXL1, and IDH1 (Makishima et al., 2011). Interestingly, this extensive analysis of the methylome at blast phase did not uncover any SNVs in the aforementioned genes or any specific SNVs in any other epigenetic modifiers (Heller et al., 2016). This is perhaps not too surprising when the genetic complexity of BP-CML and inter patient heterogeneity is taken into account and will reflect the difficulty in trying to identify a universal approach to targeting this stage of the disease.

### BMI-1 in BP CML

As described earlier, BMI-1 is a transcriptional repressor and a core component of the PRC1 complex. Bmi1 expression is normally restricted to the stem cell compartment and has been shown to be involved in the regulation and expansion of LSCs during BP-CML (Saudy et al., 2014). Compared to CP-CML, expression was shown to be further increased in aggressive forms of CML that progressed to blast phase within 3 years and during advanced phases of the disease and was shown to be 2-fold elevated in AP- and BP-CML as compared to CP, both in peripheral blood mononuclear cells and CD34^+^ cells (Mohty et al., 2007; Saudy et al., 2014). A study of 64 patients with CP-CML, with higher BMI1 expression in CD34^+^ cells at diagnosis conferred a poorer prognosis and decreased survival than those with lower levels (Mohty et al., 2007). Overall, higher BMI1 expression appears to be predictive of poorer outcome in patients with CML. BMI1 may prove to be an interesting target in attempting to eradicate more advanced phase of the disease in these patients. Recently, early phase clinical trials have begun to look at the utility of BMI-1 inhibitors in advanced solid tumors (clinicaltrials.gov #NCT02404480), gliomas (clinicaltrials.gov #NCT03605550), and for relapsed/refractory AML (clinicaltrials.gov #NCT03761069). An additional polycomb group protein, SUZ12 has also been shown to be overexpressed in bone marrow CD34^+^ cells from BP-CML patients, resulting from the activation of the non-canonical Wnt pathway (Pizzatti et al., 2010).

## Conclusion

**FIGURE 3:**
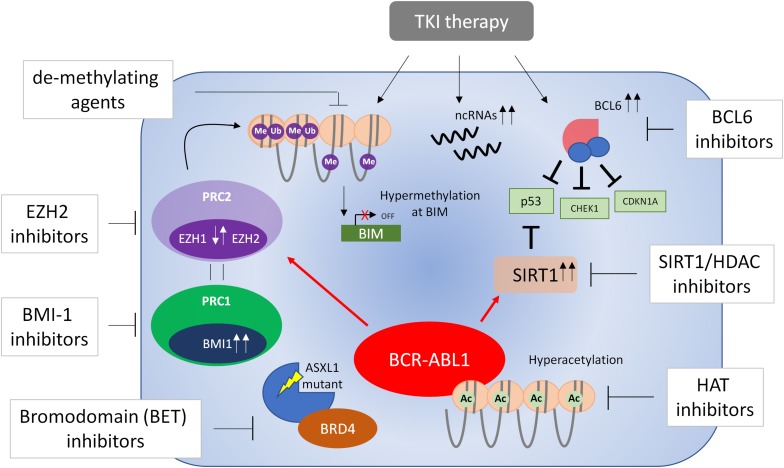
Targeting epigenetic processes in CML cells. A number of processes are involved in the epigenetic reprogramming of CML cells (see text for further details). These include dysregulation of EZH1 and EZH2, BCL6, BMI1, and SIRT1, DNA methylation, non-coding (nc)RNAs and mutations in ASXL1 – all of which are believed to contribute to the survival of bulk CML cells/LSCs or disease progression. In some cases, this dysregulation occurs only in the presence of TKI treatment. Currently there is no evidence of dysregulation of EZH1/2 in blast phase CML and therefore EZH1/2 inhibitors have not been tested in this respect. Evidence from blast phase cell lines suggests BCL6 may be a survival factor and thus BCL6 inhibitors may be effective in the treatment of accelerated and blast phase disease. With this knowledge in mind, a number of epigenetic therapies have been proposed that target these processes and may result in eradication of CML cells in combination with TKI therapy.

Whilst the development and pathogenesis of CML is well-defined in a genetic context, the ever-growing importance of epigenetic reprogramming at various stages of CML progression and in response to therapy is only now being recognized. Further understanding of epigenetic processes is required to overcome many clinical issues which still exist in CML, such as the optimal treatment of patients in more developed stages of the disease, the prerequisites for treatment discontinuation, and the factors involved in the survival of LSCs. Evolving technologies such as whole-genome sequencing, single-cell RNA-seq (Giustacchini et al., 2017), and genome-wide DNA methylation will aid in the further development of the role of epigenetics in CML, and the promise of epigenetic therapies. Furthermore, the requirement for epigenetic therapies may be considerably more important in older patients with CML due to the aberrant epigenetic processes that occur with aging (Pal and Tyler, 2016). A number of epigenetic therapies such as SIRT1 inhibitors, HDAC inhibitors and EZH2 inhibitors are currently in clinical trials for many cancer indications, and have shown promise in murine models and good safety profiles in Phase I healthy individuals. This suggests that similar trials may ensue for CML based on rationales for epigenetic therapies (Figure 3) proposed here.

## Author Contributions

JB, RK, MS, and DV wrote and reviewed the manuscript.

## Conflict of Interest Statement

The authors declare that the research was conducted in the absence of any commercial or financial relationships that could be construed as a potential conflict of interest.
